# Sensitivity of *Mycobacterium leprae* to Telacebec

**DOI:** 10.3201/eid2803.210394

**Published:** 2022-03

**Authors:** Ramanuj Lahiri, Linda B. Adams, Sangeeta Susan Thomas, Kevin Pethe

**Affiliations:** US Department of Health and Human Services, Health Resources and Services Administration, Health Systems Bureau, National Hansen's Disease Program, Baton Rouge, Louisiana, USA (R. Lahiri, L.B. Adams);; Lee Kong Chian School of Medicine, Nanyang Technological University, Singapore (S.S. Thomas, K. Pethe)

**Keywords:** Hansen disease, tuberculosis and other mycobacteria, bacteria, *Mycobacterium leprae*, leprosy, telacebec, QcrB inhibitors, terminal oxidase

## Abstract

The treatment of leprosy is long and complex, benefiting from the development of sterilizing, rapidly-acting drugs. Reductive evolution made *Mycobacterium leprae* exquisitely sensitive to Telacebec, a phase 2 drug candidate for tuberculosis_._ The unprecedented potency of Telacebec against *M. leprae* warrants further validation in clinical trials.

Leprosy, also known as Hansen disease, is a chronic infectious disease caused primarily by *Mycobacterium leprae* and to a lesser extent by *M. lepromatosis* bacteria. Both species have a strong tropism for the Schwann cells; infection causes peripheral neuropathy, which leads to the characteristic deformities and disabilities. Despite successful implementation of multidrug therapies for the treatment of leprosy, >200,000 new cases were reported globally in 2019. Drug-resistant *M. leprae* strains, although rare, are emerging in several parts of the world (*1*). Therefore, newer rapidly acting bactericidal, orally bioavailable drugs are required to shorten treatment time and reduce transmission.

The high potency of drugs targeting the cytochrome *bcc:aa_3_* terminal oxidase (also known as QcrB inhibitors) against *M. ulcerans* has been reported (*3*). Of particular importance is the finding that a single dose of the drug candidate, Telacebec (Q203) (*3*), eradicates infection in a mouse model of Buruli ulcer (*4*). The potency of drugs targeting the cytochrome *bcc:aa_3_* terminal oxidase against *M. ulcerans* is explained by the absence of a functional cytochrome *bd* oxidase, an alternate terminal oxidase that limits the potency of telacebec in *M. tuberculosis* (*5*,*6*). Like *M. ulcerans*, *M. leprae* has lost the genes encoding the cytochrome *bd* oxidase and any other alternate terminal electron acceptors (*7*). Because *M. leprae* relies exclusively on the cytochrome *bcc:aa_3_* terminal oxidase for respiration, Scherr et al. hypothesized that telacebec and related QcrB inhibitors could represent a new class of bactericidal drugs for leprosy (*2*).

The potency of telacebec was initially tested against extracellular *M. leprae* using a radio-respirometry assay to determine bacterial β-oxidation rate. This assay is used to assess viability of noncultivable *M. leprae* and measures cumulative production of CO_2_ by the bacilli when palmitic acid is the sole carbon source (*8*). Telacebec at a concentration of 0.2 nM inhibited ≈90% (p<0.001) and 2 nM inhibited ≈99.9% (p<0.0001) of *M. leprae* metabolic activity after 3 days of incubation (Figure, panel A). In comparison, rifampin used at 2.0 μM inhibited only ≈45% (p = 0.020) of the metabolic activity compared with untreated control in the same time frame (Figure, panel A). We observed a similar trend after 7 days of incubation (Figure, panel A); 0.2 nM of telacebec was significantly more potent than 2 μM of rifampin at all tested concentrations in this assay. Telacebec was also active against intracellular *M. leprae* maintained in murine bone marrow–derived macrophages (*9*). Telacebec at 2.0 nM inhibited ≈97% (p<0.001 vs. untreated) of the metabolic activity of intracellular *M. leprae* in 3 days. Telacebec was also marginally potent against intracellular *M. leprae* at 0.2 nM but required longer incubation; we observed a statistically nonsignificant reduction of ≈33% (p = 0.069) after 3 days’ incubation and a significant reduction of ≈40% (p = 0.034) after 7 days. Under similar experimental conditions, rifampin at 2.0 μM inhibited metabolic activity of intracellular *M. leprae* by ≈44% (p = 0.025) at day 3 and ≈72% (p<0.001) at day 7 compared with the untreated control group (Figure, panel B). Telacebec at 2 or 20 nM was more potent than rifampin in this assay as well.

**Figure:**
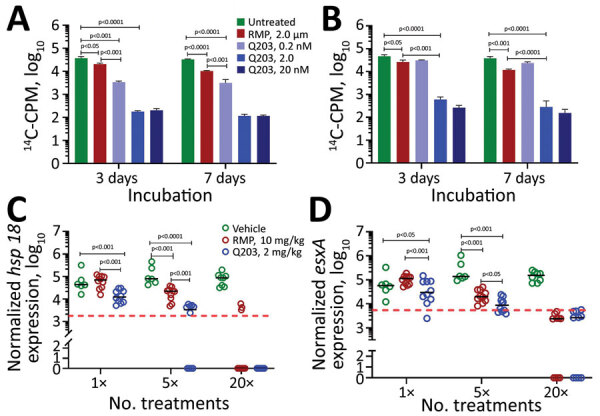
Efficacy of telacebec against *Mycobacterium leprae* bacteria in axenic culture (A), in murine bone marrow–derived macrophages (B), and in athymic nude mouse foot pad model (C, D). *M. leprae hsp18* (C) and *esxA* (D) expression levels were used as a surrogate measure of viability. For panels A and B, the assays were performed in triplicate for each condition. For panels C and D, each foot pad is taken as a data point, and the red dotted lines indicate ≈99% *M. leprae* kill. Significance was determined by 2-tailed unpaired Student *t*-test. ^14^C, carbon 14; CPM, counts per minute; Q203, telacebec; RMP, rifampin.

The high nanomolar potency of telacebec against both intracellular and extracellular *M. leprae* prompted us to evaluate its efficacy in a mouse foot pad model of infection. We inoculated groups of 5 athymic nude mice with 3 × 10^7^ viable *M. leprae* in both hind foot pads. At 8 weeks postinfection, we administered telacebec (2 mg/kg) or rifampin (10 mg/kg) by gavage as 1 dose, 5 consecutive daily doses, or 20 doses (5 days × 4 weeks). We harvested foot pads 4 weeks after completion of the drug treatment. Because *M. leprae* is noncultivable, we measured mycobacterial load using an established molecular method (*10*). We determined *M. leprae hsp18* and *esxA* expression levels as a surrogate measure of viability (*10*). Bacterial *hsp18* and *esxA* expression were significantly lower in mice receiving 1 (p<0.001) or 5 (p<0.001) consecutive doses of telacebec compared with rifampin or to the vehicle-treated control group, indicating a faster in vivo bactericidal efficacy of telacebec (Figure, panels C, D). Although >5 consecutive doses of rifampin were needed to detect a bactericidal efficacy, 1 dose of telacebec at a low dose of 2 mg/kg was sufficient to reduce the bacterial viability substantially (Figure, panels C, D).

This study demonstrates the exquisite sensitivity of *M. leprae* to telacebec and the potential of a shorter treatment regimen. Dose-finding studies in animals will help to determine an optimum dosing regimen for rapid bacterial eradication. Combination therapies between telacebec and first- or second-line drugs such as rifampin, clofazimine, or minocycline should be evaluated in preclinical animal models to guide the development of a potent, fast-acting, sterilizing drug combination for humans that has a low propensity for resistance development for humans. The curative promise of telacebec or other advanced QcrB inhibitors should be validated in human clinical trials.
